# Social Networks, the ‘Work’ and Work Force of Chronic Illness Self-Management: A Survey Analysis of Personal Communities

**DOI:** 10.1371/journal.pone.0059723

**Published:** 2013-04-02

**Authors:** Ivaylo Vassilev, Anne Rogers, Christian Blickem, Helen Brooks, Dharmi Kapadia, Anne Kennedy, Caroline Sanders, Sue Kirk, David Reeves

**Affiliations:** 1 Centre for Primary Care, Institute of Population Health, Collaboration for Leadership in Applied Health Research (CLAHRC), The University of Manchester, Manchester, United Kingdom; 2 Faculty of Health Sciences, University of Southampton, Southampton, United Kingdom; 3 School of Nursing, Midwifery and Social Work, University of Manchester, Manchester, United Kingdom; Tehran University of Medical Sciences, Iran (Republic of Islamic)

## Abstract

Self-management support forms a central aspect of chronic Illness management nationally and globally. Evidence for the success of self-management support has mainly focussed on individually-centred outcomes of behavioural change. While it is recognised that social network members play an important role there is currently a gap in knowledge regarding who provides what type of support and under what circumstances. This is relevant for understanding the division of labour and the meeting of needs for those living with a long-term condition. We therefore took a network approach to explore self-management support conceptualising it as types of illness ‘work’ undertaken within peoples’ social networks. 300 people from deprived areas and with chronic illnesses took part in a survey conducted in 2010 in the North West of England. A concentric circles diagram was used as a research tool with which participants identified 2,544 network members who contributed to illness management. The results provide an articulation of how social network members are substantially involved in illness management. Whilst partners and close family make the highest contributions there is evidence of inputs from a wide range of relationships. Network member characteristics (type of relationship, proximity, frequency of contact) impact on the amount of illness work undertaken in peoples’ networks. In networks with ‘no partner’ other people tend to contribute more in the way of illness related work than in networks with a partner. This indicates a degree of substitutability between differently constituted networks, and that the level and type of input by different members of a network might change according to circumstances. A network perspective offers an opportunity to redress the balance of an exclusively individual focus on self-management because it addresses the broader set of contributions and resources available to people in need of chronic illness management and support.

## Introduction

As part of a ‘care transition’ self-management support policies for long-term conditions (LTCs) are designed to enhance peoples’ self-management capacities and reduce the fiscal burden on health care systems [1], [2]. This has necessitated the reconfiguration of organisational interfaces, introduction of new technologies, and re-consideration of the work that stems from chronic illness. In policy terms [3] the work related to chronic illness has been divided across three (epidemiological) levels: *case management* is for patients with multiple complex conditions, involving intensive proactive professionally delivered care to avoid complications and admissions; *disease management* is for patients at some risk who are managed through guideline-based programmes in primary care; and *self-care support* refers to the longer term and more mundane work for those considered at ‘low risk’ (70–80% of those with LTCs). Interventions designed to support self-care have for the most part focused on achieving individually-centred outcomes and psychological mechanisms of behavioral change [3], [4], [5], [6], [7], [8], [9]. Whilst the literature on informal care points to the contribution made by significant others to the care of those with a chronic condition and of those requiring intensive support, less attention has been focused on developing an understanding of the division of labour, and the relational activity involved in mobilizing resources and the type of work of others implicated in self-care support in wider social contexts and outside of formal health care settings [10]. Here we look to the development of an approach capable of in-depth exploration of the nature and capacity of support provided by others [10], [11].

A broader re-conceptualisation of inter-personal relationships and illness-relevant practices requires a method that goes beyond simply increasing the number and range of contextual variables that might influence individual behavior. The work of Christakis and Fowler [12] illuminate the importance of network dynamics in the spread of health related phenomena such as giving up smoking, obesity and happiness [13], [14], [15]. Redressing the balance between individual and wider social influences in the management of health conditions is to bring into view a conceptualization of social support and of network ties as a set of relational activities, and as processes where different resources “flow” from one person to another [16]. Pescosolido and colleagues have previously theorised the interplay between illness and social networks through the Network Episode Model (NEM) [17]. Drawing on the NEM they have illuminated the activation of selected network ties which extends beyond an individuals’ capacity for coping or managing and tracks the dynamics and change of such networks over time [18].

In building on this foundational agenda it is relevant to recognize that the meaning and utility of social networks are multifaceted. Thus the term ‘social networks’ has been deployed as a metaphor to describe constellations of illness-relevant relationships and as a set of techniques for data collection and analysis. Social network mapping techniques have been applied to studies of the family [19], ageing [20], friendship [21], access to health services [22], [23], [24] and had a growing influence in the area of health and illness [22], [25], [26] being used, for example, to explore *how* social ties support or are deleterious to health [27], [28], [29]. However, with the exception of mental health (see above) within the context of chronic illness management, social network analysis is currently underdeveloped, and tends to be limited to a focus on dyadic relationships, whilst the structural properties and characteristics of the social network are often conflated with other variables such as access to social support [30]. Within this context, there is utility in focusing on the illness *work* undertaken by social network members, and on studying the patterns of the relationships and the division of labour across a range of illness-relevant practices. The latter enables an extended view beyond a focus on individual behavior.

In this paper we focus on the nature of the self-management workforce by describing constellations of illness-relevant relationships and network structural properties (network size, density, degree of fragmentation). We do this through identifying and describing the members who make up the social networks (or personal communities) of individuals. Illness ‘work’ can be understood as the visible and invisible activities that are relevant for the management of LTCs. A traditional distinction is one made between illness work, everyday work and biographical work [31], [32]. Building on previous conceptualisations here we use three different domains of chronic illness work:

- *Illness (specific) work* refers to the work related to: taking medications; regimens of taking and interpreting measurements; understanding symptoms; and making appointments.
- *Everyday work* refers to: the tasks of housekeeping and repairing; occupational labour; child rearing; support and activities related to diet and exercise, general shopping and personal care.
- *Emotional work* refers to the work related to comforting when worried or anxious about everyday matters, including health, well-being and companionship. It also includes a biographical dimension associated with the reassessment of personal expectations, capabilities and future plans, personal identity, relationships and biographical events.

Using a combined approach of exploring contributions of social network members to chronic illness work, we address three key questions:

- Who and how are members of the social networks of people with LTCs involved in the management of chronic illness?
- How does the amount and distribution of chronic illness work differ between networks where a partner/spouse is present, and those without a partner/spouse?
- How do the demographic characteristics of people with chronic illness and of their social networks relate to the nature and amount of work done?

## Methods

### Ethics Statement

All participants gave informed written consent to take part in the study. Ethical approval was obtained from the Greater Manchester Research Ethics Committee in February 2010 (ref: 10/H1008/1). All participants received £10 gift vouchers as a compensation for their time and effort.

### Design and Sample Characteristics

Participants for the study were recruited from 19 GP practices located predominantly in economically deprived areas of Greater Manchester in the North West of England and surveyed between April 2010 and January 2011. Patients with chronic heart disease (CHD) or diabetes were randomly selected from the disease registers of the consenting GP practices. Invitation letters were sent from GP practices and patients returned reply slips agreeing to be contacted by a researcher to arrange to take part in the study. Researchers contacted the patients to arrange a convenient interview time and posted out a self-complete questionnaire. Network data aiming to map the personal communities of the respondents was then collected via face-to-face interviews in participants’ homes or a place convenient to them where written consent to take part in the study was obtained. The interviews were conducted by nine researchers all of whom were closely involved with the development of the questionnaire and with the discussions during and after the pilot stage. In order to further reduce the possibility for interviewer variation in the network part of the questionnaire an interview procedure was agreed, all interviewers followed a standardised data collection protocol, and were given feedback after conducting their first interview. Interpreters were made available for participants whose first language was not English.

In total, 2,001 letters were sent and 314 reply slips were returned (15.69% response rate). 14 participants were excluded from the analysis because they did not have a full dataset (postal and network interview), and the final analysis included 300 participants (for missing data see Appendix S1). The sample was not intended to be representative as our aim was to reach a highly deprived population.

Data collection was preceded by a pilot stage of the study, where semi-structured interviews were conducted with 11 people recruited in the Greater Manchester area. The pilot interviews were administered face-to-face with the participant, they were audio-recorded, transcribed and analysed. The pilot stage informed the design of the final questionnaire and the procedure used in the process of data collection.

### Measures Used in the Study

#### The naming and placing of network members

Participants were asked to map social network members using a diagram consisting of three concentric circles [33]. In response to the question, “Who do you think is most important to you in relation to managing your condition?” network members placed in the central circle were those considered most important, members placed in middle circle were considered less important than those in the central circle, and members in the outer circle were considered less important than the two inner circles (see Appendix S2). Participants were allowed to place as many network members as they wanted, of any type of relationship they considered relevant (e.g. family, friends, medical professionals, pets), including groups and services (e.g. workplace, religious group, food delivery service) as well as individuals.

This technique of data collection (known as the name generator approach) is considered superior to approaches such as the role relation approach, which uses general relational categories and asks questions of the format, “Can you tell me what your family do for you?”, as data pertaining to each individual network member is collected which is otherwise lacking. The face-to-face interviews also provided an opportunity - compared to a postal questionnaire - for additional but initially overlooked network members to become visible during the discussion and to be included in the analysis, and for detailed information to be collected about key attributes of each network member and the contributions they make to different sets of illness-relevant tasks.

### Social Network Dimensions of Chronic Illness Management Relationships

Participants were asked about characteristics of each network member they identified as being important to the management of their chronic condition. This included: age, gender, relationship to the participant, number of years known, how far away they lived, how often they were in contact, how long they spent together when in contact, and by what means they were in contact. Each network member was coded into one of eight types based on their relationship to the participant: partner/spouse, close family (parents, children (note that these were mostly adult children) and their partners, grandchildren, siblings), other family (all other relatives not included under close family), friends (friends, neighbours, and colleagues), health professionals (GPs, nurses, pharmacists), groups (e.g. church or social group), pets, ‘other’ (e.g. food delivery organisations, care workers, etc). From this, we also counted the number of different relationship types present in each person’s network. The *perceived* links between each pair of network members (as being very close, close, or not close) were also collected and were subsequently used to create network measures of fragmentation (the extent to which network members belonged to different subgroups, calculated in relation to potential and actual number of subgroups) and density (the extent to which network members were connected to each other, calculated in relation to potential and actual ties between network members) [34]. Additional measures of each person’s social network/networking were the size of the support network (the number of members with a non-zero score for at least one work dimension); the amount of support the participant themselves gave to others in the past month (a count out of seven kinds of possible support); and – as measures of access to network resources and social capital - a score on a resource generator [35], social involvement (score across 14 possible types of involvement), and satisfaction with social involvement.

### Measuring Member Contributions to Each Work Domain

To quantify the contribution made by each network member, we devised a questionnaire consisting of 13 items addressing different aspects of the 3 domains of work (see Appendix S3 for details). Participants were asked to rate each network member on a Likert scale (1: not at all, 5: a lot) according to perceived contribution to each aspect of work. These ratings were then summed across the items in each domain to obtain a total score for each network member on each type of work. We then rescaled these scores to range from 0 (does not help at all in any aspect) to a possible maximum of 10 (helps a lot in all aspects).

### Socio Demographic and Health Measures

We collected socio-demographic characteristics of respondents that included age, gender, ethnicity, income, household tenure (own/mortgaged or rented), living arrangements, marital status, qualifications, occupational class, and employment status. We used the SF12v2 and applied structural equation modelling to obtain oblique (correlated) physical and mental component scores [36]. The two scores correlated very highly (r = 0.83), therefore we used the physical component score as a measure of perceived health status and excluded the mental component score. As a proxy for objective health status we used number of conditions. We also used area based multiple deprivation index (IMD), and for neighbourhood safety (this is a place I enjoy living in, this is a place where neighbours look after each other) and neighbourhood amenities (this area has good local transport, this area has good leisure things for people like me, this area is well provided with health services, this area is well provided with shops, banks, and postal services) we used items from the Health Survey for England.

### Analysis Methods

To describe ‘who**’** the members of peoples’ social networks were and ‘how’ they were involved in the management of chronic illness we conducted a descriptive analysis of the characteristics of the network members exploring how the contribution of members to each work domain varied according to type of relationship to the participant. We investigated the work done in two ways: first, we examined the mean work done by individual members of each relational type; second, we examined the total work done by all members of each relational type, within a network. Thus, on average an individual close family member contributed 2.77 points to the illness work domain, but – because networks typically contained several members who were close family - close family members *all together* contributed on average 7.76 points to the illness work undertaken at a network level. Thus the network-level measure acknowledges the contribution to work made by each additional close family member. When computing network-level means we averaged over only those networks where the relational type was present.

In order to explore how the presence of a partner/spouse in the network influenced work undertaken by other network members, we analysed the amounts of work done in each of the three domains of work by each type of relationship for networks where a partner/spouse was present and networks where partner/spouse was absent.

We used univariate and multivariate multilevel regression analysis (including all variables with a univariate relationship to each type of work at a p-value of < = 0.1, and using stepwise regression with backwards elimination (at p<0.05) to arrive at a final model) to identify characteristics of (i) the participant (ego); (ii) the network members; and (iii) the social network, predictive of the contribution made by a member to each work domain. To distinguish the relative influence of ego, member, and social network factors to member work scores, we first conducted three separate analyses using the variables within each of these sub-sets, and then undertook a final analysis combining the variables from all three sub-sets into a single model. To account for the multilevel nature of the network data (members within networks) all analyses were undertaken using the Stata (v12) Xtmixed command, specifying a two-level random-effects model and maximum-likelihood estimation. Network was a random effect in the model (thus treating the networks as a random sample from a population) and all other factors were specified as fixed effects. We used the robust Huber-White sandwich estimator of variance to account for non-independence of observations within networks. The variance inflation factors for the variables in each regression were all below an acceptability threshold of 10 [37]. However, since lower levels may also cause estimation problems we conducted sensitivity analysis for multivariate analyses where variables had inflation factors of greater than 2, by repeating the analysis without the variable and examining the impact on the parameter estimates and statistical significance. The only variables where this applied were network member gender, distance from ego, and relationship to ego.

## Results

### Sample Characteristics

Men constituted 64% (n = 193), and women 36% (n = 107) of the sample (Table 1). The average age of participants was 65 (ranging from 20 to 93 years), 19% (n = 58) had diabetes, 40% (n = 120) had coronary heart disease and 41% (n = 122) had both conditions, participants had on average 2 or 3 chronic conditions (multi-morbidities). The sample was predominantly white 86% (n = 259). Over half the participants were married (55%, n = 165) and almost half were retired (49%, n = 148).

**Table 1:** Ego level descriptive analysis.

Ego characteristic	N (%)
Gender	
Male	193 (64.3%)
Female	107 (35.7%)
Ethnicity	
White	259 (86.3%)
Non-white	41 (13.7%)
Condition type	
Diabetes	58 (19.3%)
Chronic Heart Disease	120 (40.0%)
Both conditions	122 (40.7%)
Tenure	
Owns	187 (62.3%)
Rents	113 (37.7%)
Marital status	
Married	165 (55.0%)
Divorced or widowed	94 (31.3%)
Never married	41 (13.7%)
Employment status	
Long-term Sickness	52 (17.3%)
Looking after home or family	19 (6.4%)
Seeking employment	6 (2.0%)
Retired	148 (49.3%)
In paid work	62 (20.7%)
None of the above or missing	11 (3.6%)
Age	Mean = 65.3 (SD = 12.62)
Total number of conditions	Mean = 2.72 (SD = 1.21)

### Who are the Social Network Members Contributing to Chronic Illness Work?: Relationship Types, Proximity and Gender

The 300 participants included in the study identified a total of 2,544 network members contributing to long-term condition management as defined by our three types of work. 1,259 (49.5%) network members were placed in the central circle of the diagram (those considered most important), 875 (34.4%) were placed in the middle circle (those considered less important than those in the central circle), and 410 (16.1%) were placed in the outer circle (considered less important than the two inner circles). Table 2 displays the demographic data related to the chronic illness management ‘workforce’. Spouses/partners and family members (close and ‘other’ family) formed the largest group carrying out tasks in relation to chronic illness (44%, n = 1,108), followed by health professionals (24%, n = 600), then friends (21%, n = 521). Spouses/partners accounted for 7% of network membership (n = 178) and smaller groups included community and voluntary groups (7%, n = 170), pets (3%, n = 66), and other types of network members (3%, n = 81). Although family members were the largest group they nevertheless constituted less than half of all network members.

**Table 2:** Information relating to the illness management workforce.

Member characteristic	N (%)
Gender	
Male	594 (23.3%)
Female	899 (35.3%)
Not relevant (including pets, groups, health professionals)	1051 (41.3%)
Age	
Under 18	78 (3.1%)
18–40 years	364 (14.3%)
40–64 years	661 (26.0%)
65 years and over	390 (15.3%)
Not applicable	1051 (41.3%)
Network member has Diabetes, CHD or CKD?	
None	2284 (89.7%)
One conditions	221 (8.6%)
More than one condition	39 (1.7%)
Types of relationships	
Partners	178 (7.0%)
Close family	725 (28.5%)
Other family	203 (8.0%)
Friends and colleagues	521 (20.5%)
Health professionals	600 (23.6%)
Pets	66 (2.6%)
Groups	170(6.7%)
Other relationships	81 (3.2%)
Type of contact with Ego	
Face-to-face	2165 (85.1%)
Telephone	328 (12.9%)
E-mail	34 (1.3%)
Other internet resources	17 (0.7%)
Geographical distance from Ego	
Co-habiting	397 (15.6%)
Short walk	751 (29.5%)
Short drive or bus journey (up to one hour)	1182 (46.5%)
Longer Journey (more than one hour)	214 (8.4%)
Frequency of contact with Ego	
Everyday	807 (31.7%)
At least once a week	764 (30.0%)
At least once a month	393 (15.4%)
At least once every couple of months	192 (7.5%)
Less often	388 (15.3%)
How long do they spend with the ego when they meet	
Up to 30 minutes	1032 (40.6%)
Between 30 minutes and 1 hour	314 (12.3%)
Between 1 and 2 hours	271 (10.7%)
More than 2 hours	927 (36.4%)

Participants generally reported high levels of contact with the people in their networks. Almost two-thirds of network members had at least weekly contact with participants (62%, n = 1571, most contact was face-to-face (85%, n = 2165), and over one third (36%, n = 927) spent over two hours with the participant at each encounter. Most network members lived in close proximity (co-habiting or within a short drive) to the respondent (92%, n = 2330). There were more female network members (35%, n = 899) than male (23%, n = 594), although around 41% (n = 1051) of all members were gender-neutral (e.g. pets, groups, health professionals).

### How are Network Members Involved in Different Types of Work?

Table 3 compares the contributions made by network members of each relational type to each work domain, in terms of both member mean scores and network-level scores. The results in table 3 are unadjusted for other ego, member or network characteristics. For all three domains, mean work scores differed significantly (p<0.001) amongst the relationship types, at both the member- and network-levels.

**Table 3:** Member and network-level mean work scores by relationship type.

	N of members	Mean (SE) member score^1^	p-value^3^	N of networks (out of 300)	Mean (SE) network-level score^2^	p-value^3^
***Illness work***						
Partner or spouse	178^4^	6.58 (0.23)		177	6.66 (0.24)	
Close family	725	2.77 (0.13)		235	7.76 (0.45)	
Other family	203	1.97 (0.20)		93	4.00 (0.55)	
Friends/colleagues	521	1.60 (0.10)		192	3.44 (0.27)	
Pets	66	0.69 (0.14)	p<0.001	56	0.73 (0.17)	p<0.001
Health professionals	600	2.23 (0.10)		267	5.00 (0.25)	
Groups	170	0.92 (0.10)		99	1.38 (0.23)	
Other relationships	81	1.36 (0.18)		56	1.94 (0.38)	
**Total**	**2544**					
**Everyday work**						
Partner or spouse	178^4^	6.39 (0.22)		177	6.43 (0.22)	
Close family	725	1.82 (0.12)		235	5.21 (0.40)	
Other family	203	1.08 (0.16)		93	2.21 (0.41)	
Friends/colleagues	521	0.92 (0.09)		192	2.00 (0.23)	
Pets	66	1.27 (0.19)	p<0.001	56	1.41 (0.23)	p<0.001
Health professionals	600	0.83 (0.75)		267	1.88 (0.17)	
Groups	170	0.89 (0.13)		99	1.37 (0.31)	
Other relationships	81	0.82 (0.15)		56	1.23 (0.28)	
**Total**	**2544**					
**Emotional work**						
Partner or spouse	178^4^	8.03 (0.19)		177	8.16 (0.21)	
Close family	725	5.00 (0.16)		235	14.63 (0.74)	
Other family	203	3.92 (0.22)		93	8.49 (0.85)	
Friends/colleagues	521	3.47 (0.16)		192	8.59 (0.60)	
Pets	66	4.15 (0.33)	p<0.001	56	4.79 (0.52)	p<0.001
Health professionals	600	1.53 (0.12)		267	3.49 (0.26)	
Groups	170	2.79 (0.21)		99	4.85 (0.58)	
Other relationships	81	1.91 (0.27)		56	2.78 (0.56)	
**Total**	**2544**					

1 Mean score for members of each relational type.

2 Combined (summed) score for members within a network, as a mean across networks that included the type.

3 Overall test of differences in scores between relational types.

4 One network included two partners/spouses.

Analysis of mean member scores by type of relationship to the participant (table 3) indicates that spouses/partners were perceived to be undertaking the largest amounts of work in all domains, with members who were close family being rated second, but with much lower mean scores. Health professionals made the third highest mean contribution to illness work, but had considerably lower ratings with respect to everyday and emotional work. ‘Other’ family members contributed substantially less than close family to all domains of work, and their mean scores were much closer to those of friends and colleagues than to those of close family.

**Figure 1:**
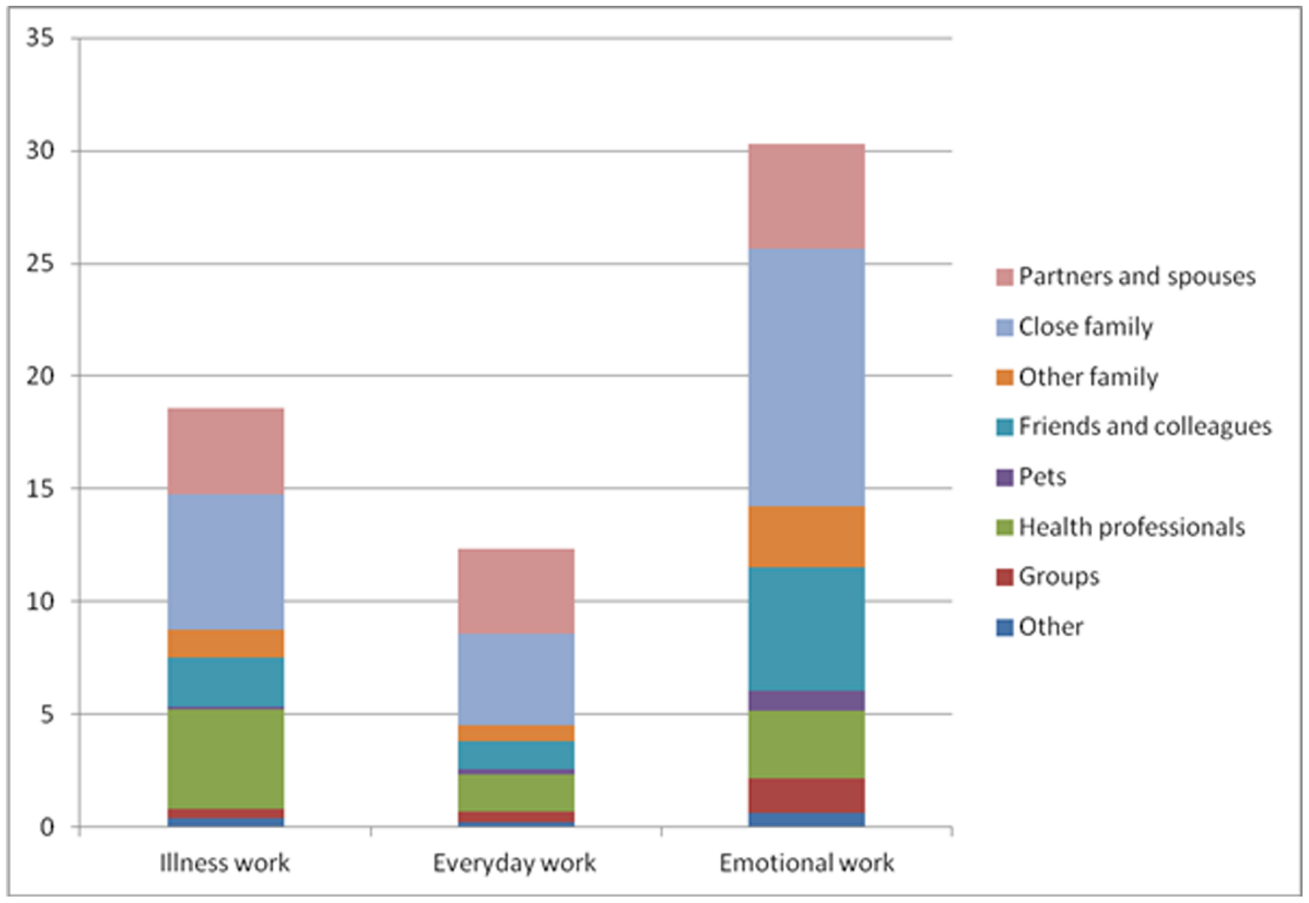
Mean network-level work scores by relationship type. The figure represents visually the differences between the mean levels for illness, emotional and everyday work and in relation to type of relationship.

The above rank orders, however, changed in several respects when considering the network-level contributions made by members of each relationship type (Table 3; Figure 1). On this measure, close family members made the highest contribution to illness work and emotional work, in particular greatly exceeding all other relationship types with respect to emotional work. Friends and colleagues made the second largest contribution to emotional work, followed by other family, with partners ranked fourth. The highest total amount of chronic illness work per network (Figure 1) was for emotional work and the lowest total amount was for everyday work. On all domains, partners and close family accounted for more than 50% of all the work done. Non-family members made the highest relative contribution to emotional work.

### How do Networks that Include a Partner/Spouse Compare to Networks Without a Partner/Spouse?

The markedly higher individual contributions made by partners/spouses to all three work domains has been established in the analyses above. To explore further the impact of partners/spouses (or more specifically the impact of their absence) on the work done by social networks we have examined amounts of work separately for networks with, and without, a partner/spouse present. Table 4 gives, for each type of relationship: (1) the mean work scores for (a) spouse networks and (b) no-spouse networks; (2) network-level work scores for (a) spouse and (b) no-spouse networks; and (3) in each case the difference between spouse and no-spouse scores. Table 4 also reports the results of statistical comparison tests between the two types of network, in terms of both the mean work score for individual network members, and the total work summed across all members. Comparisons are made, first excluding partners/spouses from the spouse networks to directly compare levels of work done by other kinds of members, then including partners/spouses to compare the networks as a whole.

**Table 4:** Member and network-level mean work scores by relationship type, broken down by networks with and without a partner/spouse.

	N of members		Mean (SE) member score			N of networks N (%)		Mean (SE) network-level score		
	No spouse	Spouse	No spouse (SE)	Spouse (SE)	Difference	No spouse	Spouse	No spouse (SE)	Spouse (SE)	Difference
***Illness work***										
Partner or spouse	–	178	–	6.46 (0.23)	–	–	177	–	6.51 (0.24)	–
Close family	274	451	3.22 (0.25)	2.49 (0.15)	0.73	91	144	8.39 (0.82)	7.31 (0.52)	1.08
Other family	84	119	2.52 (0.38)	1.56 (0.19)	0.96	38	55	5.59 (1.14)	3.00 (0.47)	2.59
Friends/colleagues	266	255	1.90 (0.19)	1.34 (0.11)	0.56	88	104	4.50 (0.47)	2.57 (0.26)	1.93
Pets	24	42	0.97 (0.19)	0.51 (0.19)	0.46	23	33	0.80 (0.19)	0.61 (0.27)	0.19
Health professionals	232	368	2.19 (0.18)	2.24 (0.12)	−0.05	101	166	4.95 (0.48)	5.00 (0.28)	−0.05
Groups	80	90	1.00 (0.16)	0.90 (0.14)	0.1	44	55	1.48 (0.41)	1.34 (0.23)	0.14
Other relationships	49	32	1.31 (0.25)	1.52 (0.26)	−0.21	34	22	2.19 (0.57)	1.95 (0.43)	0.24
**Overall**, excluding partner/spouse	**1009**	**1357**	**2.0** 1 **(0.15)**	**1.78** 1 **(0.10)**	**0.23 p = 0.18**	**123**	**177**	**16.42** 2 **(1.01)**	**13.62** 2 **(0.78)**	**2.81 p = 0.022**
**Overall**, including partner/spouse	**1009**	**1535**	**2.0** 1 **(0.15)**	**2.32** 1 **(0.11)**	**−0.32 p = 0.07**	**123**	**177**	**16.42** 2 **(1.01)**	**20.12** 2 **(0.84)**	**−3.71 p = 0.005**
***Everyday work***										
Partner or spouse	–	178	–	6.37 (0.22)	–	–	177	–	6.41 (0.22)	–
Close family	274	451	1.93 (0.20)	1.75 (0.15)	0.18	91	144	5.33 (0.63)	5.13 (0.51)	0.2
Other family	84	119	1.30 (0.32)	0.91 (0.15)	0.39	38	55	2.88 (0.76)	1.72 (0.44)	1.16
Friends/colleagues	266	255	0.98 (0.15)	0.87 (0.11)	0.11	88	104	2.39 (0.39)	1.68 (0.26)	0.71
Pets	24	42	1.21 (0.32)	1.30 (0.24)	−0.09	23	33	1.20 (0.34)	1.58 (0.30)	−0.38
Health professionals	232	368	0.74 (0.12)	0.88 (0.10)	−0.14	101	166	1.68 (0.28)	1.99 (0.21)	−0.31
Groups	80	90	0.91 (0.22)	0.89 (0.16)	0.02	44	55	1.49 (0.59)	1.29 (0.28)	0.2
Other relationships	49	32	0.71 (0.17)	1.01 (0.28)	−0.3	34	22	1.25 (0.37)	1.16 (0.43)	0.09
**Overall**, excluding partner/spouse	**1009**	**1357**	**1.09** 1 **(0.19)**	**1.09** 1 **(0.08)**	**0.0 p = 0.995**	**123**	**177**	**8.92** 2 **(0.78)**	**8.33** 2 **(0.62)**	**0.59 p = 0.54**
**Overall**, including partner/spouse	**1009**	**1535**	**1.09** 1 **(0.19)**	**1.70** 1 **(0.13)**	**−0.61 p<0.001**	**123**	**177**	**8.92** 2 **(0.78)**	**14.74** 2 **(0.65)**	**−5.82 p<0.001**
***Emotional work***										
Partner or spouse	–	178	–	7.84 (0.19)	–	–	177	–	7.89 (0.20)	–
Close family	274	451	5.20 (0.27)	4.85 (0.20)	0.35	91	144	14.49 (1.23)	14.66 (0.93)	−0.17
Other family	84	119	4.48 (0.39)	3.53 (0.26)	0.95	38	55	10.63 (1.66)	7.07 (0.83)	3.56
Friends/colleagues	266	255	3.97 (0.25)	3.06 (0.20)	0.91	88	104	10.82 (0.98)	6.75 (0.68)	4.07
Pets	24	42	4.67 (0.49)	3.84 (0.44)	0.83	23	33	4.54 (0.56)	4.96 (0.78)	−0.42
Health professionals	232	368	1.75 (0.22)	1.38 (0.14)	0.37	101	166	3.89 (0.51)	3.19 (0.29)	0.7
Groups	80	90	3.23 (0.28)	2.46 (0.29)	0.77	44	55	5.98 (1.06)	4.01 (0.59)	1.97
Other relationships	49	32	2.23 (0.38)	1.65 (0.39)	0.58	34	22	3.58 (0.79)	2.16 (0.75)	1.42
**Overall**, excluding partner/spouse	**1009**	**1357**	**3.61** 1 **(0.19)**	**3.09** 1 **(0.13)**	**0.52 p = 0.024**	**123**	**177**	**28.90** 2 **(1.83)**	**23.43** 2 **(1.49)**	**5.48 p = 0.019**
**Overall**, including partner/spouse	**1009**	**1535**	**3.61** 1 **(0.19)**	**3.73** 1 **(0.13)**	**−0.12 p = 0.61**	**123**	**177**	**28.90** 2 **(1.83)**	**31.32** 2 **(1.53)**	**−2.41 p = 0.31**

1 Mean work done by a member of a network.

2 Total work done by all members of a network (mean network total).

**Figure 2:**
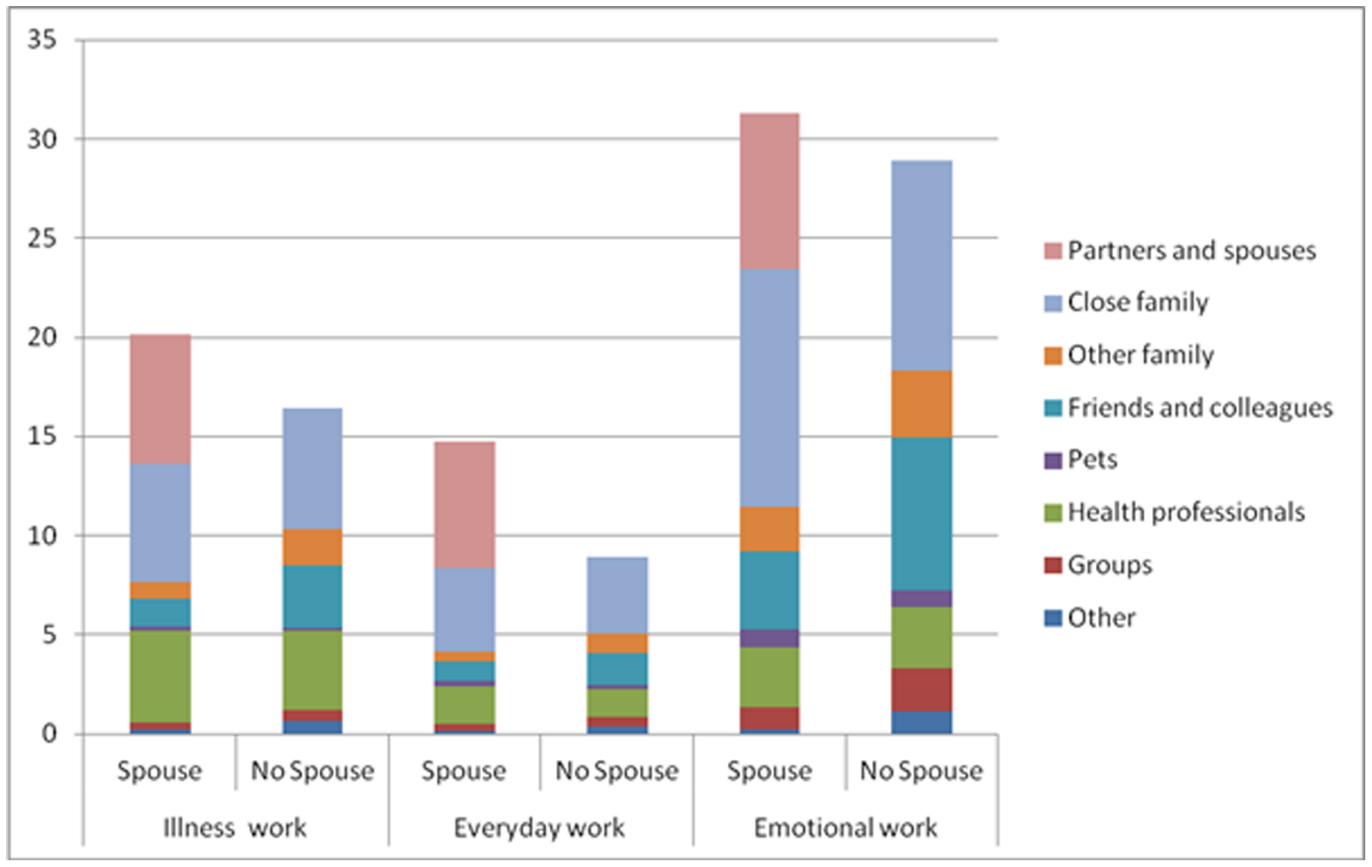
Mean network-level work scores by relationship type for networks with and without a partner/spouse. The figure compares partner and no-partner networks. The figure represents visually the differences between the mean levels for illness, emotional and everyday work and in relation to type of relationship.

Comparison of networks with and without a partner shows the average amount of work performed by non-partners does not differ significantly for the illness and everyday work domains, but that members in networks without a partner do significantly more emotional work on average (P<0.05). Mean levels of emotional work are higher for all types of members, but particularly for other family and friends/colleagues. Total amounts of work done by non-partners is significantly greater in networks without partners for illness and emotional work (p<0.05), but not for practical work. Most of this additional work is contributed by other family and friends/colleagues and to a lesser extent by groups, but not by close family. When we repeated the analysis including the contribution of partners to total work, overall amounts of illness and everyday work were significantly higher in networks with spouses (p<0.01 and p<0.001 respectively), but levels of emotional work did not differ. Taken together, these results indicate that other network members to some degree compensate for the absence of a spouse/partner, but that overall levels of work in networks with a spouse/partner are nonetheless generally higher than in networks without a partner/spouse. These results can be seen visually in Figure 2.

### What Ego and Social Network Dimensions Influence the Type and Amount of Work Undertaken?

**Table 5:** Univariate and multivariate analysis of work done by network members: Ego characteristics.

	Illness Work				Everyday Work				Emotional Work			
	Univariate		Multivariate		Univariate		Multivariate		Univariate		Multivariate	
*Ego characteristics*	Co-eff	P-value	Co-eff	P-value	Co-eff	P-value	Co-eff	P-value	Co-eff	P-value	Co-eff	P-value
Age	.011	.14			−.013	.03			.002	.78		
Gender (Female)	−.008	.97			−.261	.06			.186	.43		
Ethnicity (non-white)	.155	.57			.550	.03	.634	.01	−.046	.89		
N of conditions	.153	.05	.153	.05	−.029	.60			.146	.10		
SF12 PCS (higher score denotes better health)	−.015	.09			.006	.39			−.002	.85		
House tenure (rents)	−.170	.34			−.278	.04			.149	.51		
Qualifications (higher score denotes higher qualifications)	−.013	.80			.027	.54			−.070	.29		
Income (higher score denotes higher income)	−.100	.84			.104	.01	.122	.001	.017	.79		
Occupational class (based on NS-SEC categories, 1 highest,7 lowest)	.051	.21			.025	.44			.114	.02	.100	.049
Deprivation (higher IMD score indicates higher deprivation)	.002	.74			−.001	.59			.002	.75		
Neighbourhood- amenities (higher score indicates highersatisfaction with amenities)	.105	.23			.090	.21			.306	.005	.285	.01
Neighbourhood –safety (higher scores denotes higherperception of safety)	.028	.79			.025	.76			.134	.28		
	**Within networks**		**0%**		**Within networks**		**0%**		**Within networks**		**0%**	
**R-squared**	**Between networks**		**1.1%**		**Between networks**		**5.2%**		**Between networks**		**3.3%**	
	**Total**		**0.5%**		**Total**		**1.3%**		**Total**		**1.9%**	

#### Ego characteristics (table 5)

Few significant relationships were found between characteristics of the participants and the levels of work done by individual members of their networks. After multivariate control, only ethnicity (p<0.01) and income (p<0.001) were significantly related to level of everyday work undertaken: non-white and more affluent participants on average received slightly higher amounts of everyday work. Members on average did more illness work for participants with more co-morbidities, and levels of emotional work were higher for participants in lower occupational classes or in more highly-rated neighbourhoods. However, these relationships explained only small amounts of the differences in levels of support between participants (no more than 5% of the variability in any form of work).

We note that participant age and gender were unrelated to member work levels in any domain after multivariate control: a first-order negative relationship between increasing age and everyday work ceased to be significant after control for ethnicity and income.

#### Member characteristics (table 6)

**Table 6:** Univariate and multivariate analysis of work done by network members: member characteristics.

	Illness Work				Everyday Work				Emotional Work			
	Univariate		Multivariate		Univariate		Multivariate		Univariate		Multivariate	
*Member characteristics*	Co-eff	P-value	Co-eff	P-value	Co-eff	P-value	Co-eff	P-value	Co-eff	P-value	Co-eff	P-value
Gender (compared to Male)	–		–		–		–		–		–	
Female	.787	<.001	.590	<.001	.743	<.001	.496	<.001	.654	<.001	.466	<.001
Not applicable	−.644		.180		−.793		.193		−2.30		.084	
Proximate child	1.02	<.001	.855	<.001	.817	<.001	.565	.004	2.12	<.001	.933	<.001
Contacts Frequently	1.23	<.001	.846	<.001	1.58	<.001	.711	<.001	3.19	<.001	1.76	<.001
Distance (compared to Co-habits)	–		–		–		–		–		–	
Within short walk	−2.54		−.858		−3.37		−1.65		−3.52		−.891	
Within short car/bus journey	−2.46	<.001	−.869	.005	−3.42	<.001	−1.71	<.001	−3.51	<.001	−.849	<.001
Further away	−2.78		−1.03		−3.59		−1.84		−2.67		−.404	
Relationship (compared partner/spouse)	–		–		–		–		–		–	
Close family	−3.82		−3.38		−4.57		−3.38		−3.03		−2.58	
Other family	−4.62		−3.45		−5.31		−3.49		−4.11		−2.73	
Friends or colleagues	−4.98		−3.90		−5.46		−3.70		−4.56		−3.25	
Pets	−5.90	<.001	−5.54	<.001	−5.12	<.001	−4.85	<.001	−3.88	<.001	−3.36	<.001
Health professionals	−4.36		−2.53		−5.56		−3.17		−6.50		−3.79	
Groups	−5.66		−4.34		−5.49		−3.55		−5.24		−3.54	
Other	−5.23		−3.84		−5.56		−3.55		−6.11		−4.22	
	**Within networks**		**39.9%**		**Within networks**		**45.1%**		**Within networks**		**50.9%**	
**R-squared**	**Between networks**		**19.6%**		**Between networks**		**28.7%**		**Between networks**		**17.4%**	
	**Total**		**34.1%**		**Total**		**41.2%**		**Total**		**38.7%**	

By contrast, all of the member characteristics demonstrated strong relationships with all three types of work. Across all three domains, higher levels of work were undertaken by network members who were: female (compared to male or gender-neutral); a proximate child; in frequent contact; cohabitants; or a partner/spouse. These factors explained high percentages (between 40% and 50%) of the variability between members within each network, and also a notable percentage (17% to 30%) of the variability from one network to another. Sensitivity analyses excluding variables with inflation factors higher than 2 (member gender, distance from ego, and relationship to ego) one at a time resulted in no changes in statistical significance, although parameter estimates were in each case closer to those found under the univariate analyses.

#### Network characteristics (table 7)

First-order relationships were observed between several network characteristics and the work undertaken by network members, for each work domain. However, after multivariate control many of these relationships ceased to be statistically significant. Individual members did higher levels of everyday work in networks that were denser (ie where more members knew one-another) or smaller in size. Higher levels of illness work were also performed in denser, and also less fragmented, networks, and when the ego had less social involvement in general. Emotional work levels were also higher in denser networks, and in addition where the relational make-up of the network was more diverse and where the ego themselves gave less support to others. These network characteristics explained small but not insubstantial percentages (7% to 14%) of the variability in member work levels between networks.

**Table 7:** Univariate and multivariate analysis of work done by network members: network characteristics.

	Illness Work				Everyday Work				Emotional Work			
	Univariate		Multivariate		Univariate		Multivariate		Univariate		Multivariate	
*Member characteristics*	Co-eff	P-value	Co-eff	P-value	Co-eff	P-value	Co-eff	P-value	Co-eff	P-value	Co-eff	P-value
Fragmentation	−.599	.02	−1.89	<.001	−.099	.62			.417	.18		
Density	.947	.03	2.84	<.001	.872	.01	0.960	.002	1.47	.006	1.72	.002
Size of support network	−.098	<.001			−.071	<.001	−.075	<.001	.057	.063		
Mix of agents	−.182	.01			−.128	.02			.176	.041	.256	.003
Social involvement	.224	.001	−.153	.02	−.119	.04			−.097	.28		
Satisfaction with social involvement (higher scorehigher satisfaction)	−.066	.46			−.042	.57			−.119	.35		
Resource generator (higher score denotes higher accessto resource)	−.002	.65			.005	.12			.005	.28		
Total support given by ego	−.266	.01			−.036	.66			−.274	.026	−.300	.011
	**Within networks**		**0.0%**		**Within networks**		**0.0%**		**Within networks**		**0.0%**	
**R-squared**	**Between networks**		**14.1%**		**Between networks**		**7.7%**		**Between networks**		**7.1%**	
	**Total**		**4.2%**		**Total**		**1.5%**		**Total**		**2.5%**	

#### Combined model (table 8)

When the ego, member and network characteristics were combined together in multivariate analysis, all of the member characteristics continued to have strong relationships with the levels of work done by individual members, but there were fewer significant associations with ego and network factors. Of these, only network size remained related to everyday work, suggesting that the previously observed relationships with ethnicity, income and network density could be accounted for principally by associations between these and the characteristics of the network members themselves. Similarly, participant social involvement ceased to have a significant relationship with illness work, and density and occupational class to be associated with emotional work. Nonetheless, the combined models explained quite considerable amounts (around 40%) of the total within-and between-network variance for all three work domains.

**Table 8:** Multivariate analysis of work done by network members: Ego, member and network characteristics combined.

	Illness Work		Everyday Work		Emotional Work	
	Multivariate		Multivariate		Multivariate	
	Co-eff	P-value	Co-eff	Co-eff	P-value	Co-eff
**Ego characteristics**						
No of conditions	.197	.001				
Neighbourhood Amenities					.294	.001
**Member characteristics**						
Gender (compared to Male)	–		–		–	
Female	.582	<0.001	.492	<.001	.467	<.001
Not applicable	.182		.182		.071	
Proximate child	.842	<0.001	.554	.005	.953	<0.001
Contacts Frequently	.833	<0.001	.728	<.001	1.75	<0.001
Distance (compared to Co-habits)	–		–		–	
Within short walk	−.888		−1.65		−.907	
Within short car/bus journey	−.897	0.003	−1.71	<.001	−.849	<0.001
Further away	−1.05		−1.83		−.404	
Relationship (compared to partner/spouse)	–		–		–	
Close family	−3.36		−3.35		−2.59	
Other family	−3.41		−3.44		−2.76	
Friends or colleague	−3.86	<0.001	−3.67	<.001	−3.26	<0.001
Pet	−5.54		−4.81		−3.38	
Health professional	−2.55		−3.14		−3.79	
Group	−4.31		−3.51		−3.55	
Other	−3.84		−3.51		−4.24	
**Network characteristics**						
Size of support network			−.044	0.006		
Mix of agents					.188	.020
Fragmentation	−1.57	<0.001				
Density	1.80	0.007				
Total support given by ego	−.210	0.009			−.325	.003
**R-squared within networks**	**39.9%**		**45.1%**		**50.9%**	
**R-squared between networks**	**29.9%**		**30.4%**		**22.3%**	
**R-squared total**	**37.3%**		**41.4%**		**41.3%**	

Of particular note is the fact that under the combined models none of the ego social status factors (i.e. income, class, education, housing, area deprivation) emerged as predictors of the work done by individual members. Similarly, with the exception of a weak relationship between degree of co-morbidity and illness work, there were no significant relationships between measures of ego health and member work levels.

## Discussion

Our starting point in this paper was to develop an approach capable of drawing on and incorporating a broad ecological and social context of health that extended beyond an individual behavioural focus to self-management support. In combining aspects of social network analysis with an extended view of support, using notions of chronic illness ‘work’, we explored the extent to which different types of relationships are implicated. Whilst previous research highlights the important role that informal sources of care play in the management of LTCs [38] the conceptualization of social networks and types of work, and the process of data collection and analysis, used in this study has helped to complement more traditional approaches [39]. Specifically the network approach adopted here moves us beyond a focus on networks as a broad metaphor for social context or reference to dyadic relationships, and is an attempt to explore the interdependencies between different relationships, network characteristics, and types of illness related work within the context of illness management.

Our analysis points to a wide range of network members who contribute substantively to chronic illness-relevant activities. The problems of chronic illness go beyond one’s own capacity for managing and network ties are called upon selectively in order to deal with such problems [18], [40]. Whilst critical moments and the ensuing biographical disruption have been identified as key catalysts for network dynamics [18] here we focussed on aspects of chronic illness trajectories that included the more mundane everyday demands of living life with a chronic condition. These aspects of living with a chronic condition extend beyond the extra ordinary circumstances of a crises or short lived entry into the sick role, which require a temporary demand and mobilisation of short-term assistance and obligations from others [41]. Chronic condition support over a longer period requires a semi-permanent increase in assistance and obligations represented by the types of chronic illness work discussed earlier. We found that most of the work undertaken for people with a chronic condition originates from family members, especially from partners and close family (such as adult children and their partners) [42]. Unsurprisingly, partners and close family tended to be involved in all types of work and the dependence on partners for carrying out activities was high. However, our results also pointed to the additional value of being able to access and mobilise a diverse set of relationships by showing how the contributions made by different kinds of members of a network varied. Whilst the contributions made by the rest of the network (distal family members and groups) were more limited compared to partners and close family members, a broader set of relationships were implicated in making contributions particularly to emotional support. This might suggest that people who are meaningfully engaged in things beyond family and friends have greater access to health-relevant support and are more accessible to interventions and possibly more able to adapt to new health practices. Weak ties in particular have been found to be relevant to help-seeking and means of accessing other networks, assisting in the diversification of information and the mobilisation and use of resources. This might be because of their reach to ‘networks of networks’ which are more open to policy interventions although, belonging to ‘networks of networks’ does not in itself guarantee improved health outcomes [26], [43]. In contrast, homogenous networks, and the dominance of strong ties, especially those of partners and close family, offer less scope for change and thus also less potential to intervention. This might be due to the high level of embeddedness of these relationships into valued social roles, a sense of responsibility to others, existing routines, unwillingness and/or inability of close others to adopt changes. This points to the value of taking into consideration the roles and interactions of *diverse networks* when considering the everyday (practical) and the emotional needs of people with a chronic condition and resonates with research in other areas of social life where diverse networks have been found to be associated with providing stable and adaptive support [23], [24], [33], [44], [45], [46].

A further relevant dimension of lay care we identified is the extent to which network members can act as substitutes for each other in providing illness-relevant work. We found that there is a degree of substitutability between relationship types although no one type of relationship is likely to be able to satisfy or substitute for a full range of needs [47]. Furthermore, our findings indicate that there are differences in the extent to which network members might act as substitutes for each other in relation to different types of work. Our findings indicate that emotional work is the type of work that is most dispersed and open to contributions from a wider set of relationships. The considerably lower total amount of everyday work undertaken by network members might suggest that this type of work is retained by people with the chronic illness themselves and that the meaning and nature of the tasks make it less amenable to transferring to others including partners. This may also indicate the high value and self-validation placed by individuals on carrying out personal, mundane and routine everyday tasks where the involvement of others is unacceptable, and therefore more frequently eschewed. Research in a different cultural context suggests shifts in perceptions of housework amongst the recipients of care in which unpaid responsibilities are viewed more as an opportunity source of fulfilment than burden [48].

We also found that after controlling for network member characteristics, levels of work undertaken were unrelated to any of the ego social status measures: the assistance given by members was the same regardless of participant income, education, occupational class, or area. Previous research suggests that whilst medical care may be distributed inversely to need, the “inverse care law” [49], informal care is more usually positively related to need, the positive care law [50]. However, we found that physical health per se (as measured by the SF12) was not associated with the average contribution of network members in any work domain. While higher numbers of comorbidities were related to illness work, the relationship was only weak and comorbidity was unrelated to everyday or emotional work (although ‘extra work’ caused by poor health may be done by specific others and could have been missed by our analysis). Rather, the main drivers of support were entirely to do with characteristics of the members themselves, most importantly: being a partner/spouse; female gender; living with or near the participant; and frequency of contact. First-order relationships to social status measures, weak in any case, could in fact be accounted for by confounding with the member profile of the networks. Also noteworthy, though not unexpected, are the considerably higher levels of work contributed by members cohabiting with the person with the LTC. This might be related to the regularity of input required and perceptions about the ability to access just in time support, and raises the question of which work could be provided as part of telecare. More generally, our findings indicate that the amount of work conducted by network members depends little on the socioeconomic and health characteristics of the person with the condition, but principally upon who the people in their network are.

Whilst limited in its remit compared to other research in adjacent health fields the social network analysis presented here has helped to elaborate the crucial elements of illness management that lie outside the confines of both the individual and traditional health service. It has also pointed to the potential of this approach for the future elaboration of social policy and implementation in the field of chronic illness management. In particular it points to the limitations of dominant health and chronic illness policy that focuses overwhelmingly on individual behavior change and the use of individual motivations and self-efficacy at the expense of collective ties, resources and responses to need in contributing to coping and managing chronic conditions effectively [51]. In this sense, this study only offers a snapshot of an approach to illness management, which needs to be further explored with a longitudinal focus on the meanings, representations, and different contexts of illness-relevant work, as well as with a focus on the broader political economy of chronic illness.

### Limitations

There are several limitations to this study. These include the relatively low response rate and the relatively small sample size. The response rate could be partly due to our focus on deprived areas and on difficult to reach groups, which has been reported in other studies. While the network data collection techniques chosen for the study offered the opportunity of collecting in-depth data on network members it is also a very labour intensive technique that requires face-to-face interviews and thus makes it difficult to achieve a large sample size. We also recognise that it is possible that the sample might be biased due to a tendency for people who are managing better to take part in research.

## Supporting Information

Appendix S1 **Missing data.**
(DOCX)

Appendix S2 **Network generating diagram.**
(DOCX)

Appendix S3 **Types of chronic illness work and questions used in the study.**
(DOCX)
