# Supplementary material for: Social Networks, the ‘Work’ and Work Force of Chronic Illness Self-Management: A Survey Analysis of Personal Communities
Source: PLoS One. 2013 Apr 2;8(4):e59723. doi: 10.1371/journal.pone.0059723 (PMC3615067; doi:10.1371/journal.pone.0059723)
Supplement: Appendix S1 — Missing data. (DOCX) [file pone.0059723.s001.docx]

**Appendix S1. Missing data.**

Rates of missing data were typically very low. For variables where the rate was less than 5% (of the 300 networks) we substituted the mode (the most common value) where the variable was categorical or ordinal in type, and the mean where it was continuous. There were six variables where the rate of missing data was greater than 5% (age, ethnicity, highest qualification, income, occupational class, neighbourhood amenities score). In these cases we used regression imputation (based on occupational Class, age at time of interview, IMD score, rent/home- ownership status, self-employed status, paid work status, supervision of other employees and ethnicity - with the relevant missing variable dropped from the list). We used logistic regression to predict ethnicity, and linear regression for other variables, rounding the prediction to the nearest catergory in the case of ordinal variables.
