# Supplementary material for: Social Networks, the ‘Work’ and Work Force of Chronic Illness Self-Management: A Survey Analysis of Personal Communities
Source: PLoS One. 2013 Apr 2;8(4):e59723. doi: 10.1371/journal.pone.0059723 (PMC3615067; doi:10.1371/journal.pone.0059723)
Supplement: Appendix S2 — Network generating diagram. (DOCX) [file pone.0059723.s002.docx]

**Appendix S2. Network generating diagram.**

YOU
