# Supplementary material for: Social Networks, the ‘Work’ and Work Force of Chronic Illness Self-Management: A Survey Analysis of Personal Communities
Source: PLoS One. 2013 Apr 2;8(4):e59723. doi: 10.1371/journal.pone.0059723 (PMC3615067; doi:10.1371/journal.pone.0059723)
Supplement: Appendix S3 — Types of chronic illness work and questions used in the study. (DOCX) [file pone.0059723.s003.docx]

**Appendix S3. Types of chronic illness work and questions used in the study.**

| **Types of work** | **Questions used** |
| --- | --- |
| Illness work | This person helps me with the day-to-day management of my long-term condition.  This person helps me when I need to re-arrange things due to health problems.  This person helps me understand advice so I know what I have to do to manage my condition.  This person helps me with things related to medications.  This person helps me organise tasks related to my condition, including arranging appointments with health care staff, getting prescriptions etc.  This person stands in for me or stands up for me when I am unwell or unable to stand up for myself.  This person comforts me when I am worried or anxious about my health problems |
| Practical work | This person helps me with the day-to-day running of my household.  This person helps me with things related to my diet.  This person helps me with things related to physical activities and exercise. |
| Emotional work | This person makes me feel good about myself.  This person helps me value and enjoy life.  This person helps me achieve personal goals. |
